# A Tri-*O*-Bridged Diels-Alder Adduct from Cortex Mori Radicis

**DOI:** 10.3390/molecules23010133

**Published:** 2018-01-09

**Authors:** An-Qi Lu, Ming-Hua Chen, Jie Gao, Lu Wang, Han-Yu Yang, Lan Li, Bo Zhang, Hao-Ke He, Su-Juan Wang

**Affiliations:** 1 State Key Laboratory of Bioactive Substance and Function of Natural Medicines, Institute of Materia Medica, Chinese Academy of Medical Sciences and Peking Union Medical College, Beijing 100050, China; anqilu@163.com (A.-Q.L.); wanglu@imm.ac.cn (L.W.); yanghanyu@imm.ac.cn (H.-Y.Y.); lilan92@outlook.com (L.L.); zhangbo@imm.ac.cn (B.Z.); hehaoke@imm.ac.cn (H.-K.H.); 2 Institute of Medicinal of Biotechnology, Chinese Academy of Medical Sciences and Peking Union Medical College, Beijing 100050, China; mingsunlight@sina.com; 3 GRU Cancer Center, Augusta University, Augusta, GA 30912, USA; jgao@augusta.edu

**Keywords:** Cortex Mori Radicis, *Morus*, Diels-Alder adduct, calculated ECD, antioxidation

## Abstract

Sanggenon X, an unusual tri-*O*-bridged Diels-Alder adduct, was isolated from Cortex Mori Radicis. Its structure was established by spectroscopic analysis, including NMR and HR-MS (High Resolution Mass Spectrometry). Sanggenon X contained three *O*-bridged rings, where the oxygenated bridgeheads were all quaternary carbons. Chemical methylation was carried out to deduce the linkages of the three *O*-bridges. The absolute configuration was determined by calculating the ECD (Electronic Circular Dichroism) using the TDDFT (Time-Dependent Density Functional Theory) method. Sanggenon X showed significant antioxidant activity against Fe^2+^-Cys-induced lipid peroxidation in rat liver microsomes, and was as effective as the positive control, curcumin.

## 1. Introduction

Cortex Mori Radicis is the root bark of some *Morus* species (e.g., *M. alba*, *M. mongolica*, *M. cathayana*, and *M. australis*), and has been used in traditional Chinese medicine as an antidiabetic, a diuretic, and an expectorant agent. Various compounds have been identified from *Morus* plants, such as Diels-Alder (D-A) adducts, stilbenes, flavonoids, and alkaloids. Their antioxidant [1,2,3], anti-inflammatory [4,5], antimicrobial [6,7,8,9], anticarcinogenic [10,11,12], and antidiabetic [13] activities have been widely reported. In our previous studies, some analgesic benzofuran-type stilbenes related to the traditional antirheumatic usage of Cortex Mori Radicis were reported [14]. Our ongoing research led to the discovery of an unusual tri-*O*-bridged D-A compound in which oxygenated bridgeheads were all quaternary carbons. Herein, we report the isolation, structure elucidation, and the absolute configuration of the previously undescribed compound named Sanggenon X (**1**).

## 2. Results and Discussion

**Figure 1:**
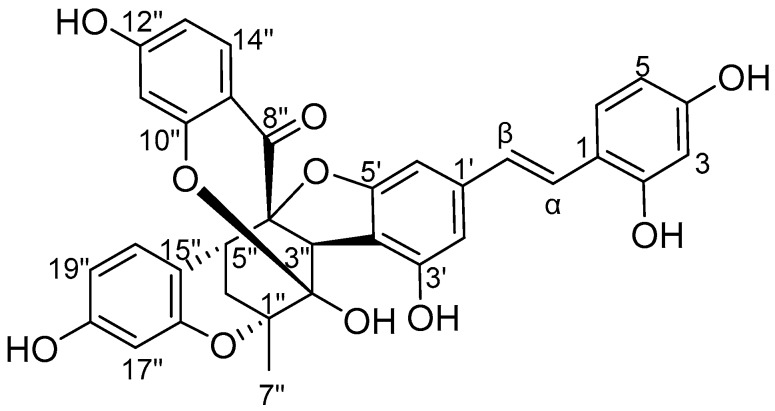
Structure of sanggenon X (**1**).

Sanggenon X (Figure 1) was obtained as a yellowish-brown amorphous powder. Its IR spectrum showed absorption bands assigned to carbonyl (1685 cm^−1^) and aromatic (1605, 1509 and 1459 cm^−1^) groups. The molecular formula C_34_H_26_O_10_ was determined by (+)-ESI HR-MS (electrospray ionization high resolution mass spectrometry) at *m*/*z* 595.1586 [M + H]^+^ (calcd for C_34_H_27_O_10_^+^, 595.1599). The ^1^H-NMR spectrum of **1** (Table 1) showed three aromatic moieties as follows: (a) a trisubstituted benzoyl at *δ*_H_ 7.44 (d, *J* = 8.7 Hz, H-14″), 6.48 (d, *J* = 8.7 Hz, H-13″), and 6.09 (s, H-11″); (b) a trisubstituted phenyl ring at *δ*_H_ 6.51 (d, *J* = 9.0 Hz, H-20″), 6.22 (d, *J* = 9.0 Hz, H-19″), and 6.23 (s, H-17″); (c) a stilbene moiety at *δ*_H_ 7.30 (d, *J* = 8.4 Hz, H-6), 6.23 (d, *J* = 8.4 Hz, H-5), 6.30 (s, H-3), 6.44 (s, H-6′), 6.16 (s, H-2′), 7.09 (d, *J* = 16.2 Hz, H-α), and 6.74 (d, *J* = 16.2 Hz, H-β). These fragments in the downfield region were similar to a known D–A adduct, kuwanon Y [15,16]. In addition, the spectrum showed five singlets assigned to active hydroxyl protons at *δ*_H_ 9.55 (OH-2), 9.38 (OH-4), 9.29 (OH-18″), 8.85 (OH-3′), and 6.64 (OH-2″). In the upfield region, there were two methines at *δ*_H_ 3.17 (s, H-3″) and 2.66 (s, H-5″); one methylene at *δ*_H_ 2.51 (d, *J* = 13.8 Hz, H-6″) and 1.77 (dd, *J* = 13.8, 3.0 Hz, H-6″); and one methyl group at *δ*_H_ 1.61 (s, H-7″). Combined with the seven aliphatic carbons *δ*_C_ 109.1 (C-2″), 91.4 (C-4″), 74.4 (C-1″), 47.3 (C-3″), 36.6 (C-5″), 30.1 (C-6″), 22.1 (C-7″) in the ^13^C-NMR spectrum, the spectroscopic data established that the structure was a methylcyclohexane D-A skeleton, as shown in Figure 1.

**Figure 2:**
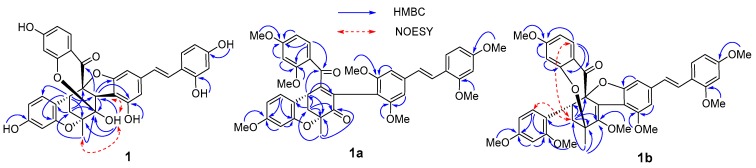
Key correlations of compounds **1**, **1a**, and **1b** in HMBC and NOESY spectra.

In the HMBC spectrum (Figure 2), the cross-peaks from methyl protons H-7″ to C-1″/C-2″/C-6″; from methine H-3″ to C-2″; from methane H-5″ to C-1″/C-3″/C-4″; and from methylene H-6″ to C-1″/C-2″/C-4″ established that the D-A skeleton was 1″,2″,4″-trioxymethylcyclohexane. The proton H-3″ was correlated with C-3′/C-4′/C-5′ (*δ*_C_ 154.2, 110.7, and 159.2) of stilbene, suggesting that the stilbene was attached to the C-3″ of the D-A skeleton at C-4′ position. The cross-peaks from H-3″/H-5″ to C-8″ (*δ*_C_ 194.9) confirmed the linkage from benzoyl C-8″ to C-4″. The correlations from H-5″ to C-16″/C-20″ (*δ*_C_ 154.6 and 133.3) showed that the phenyl group was connected at C-15″ to C-5″. The cross-peaks from an unusual active proton OH-2″ to C-2″ and C-3″, combined with the chemical shift of C-2″ (*δ*_C_ 109.1), demonstrated that the C-2″ was a hemiketal carbon. Because four phenolic hydroxyl protons (OH-2, 4, 3′, 18″) were correlated with their own adjacent carbons, there must be three oxygen-bridges connecting C-1″, C-2″, or C-4″ of cyclohexane to C-5′, C-10″, C-12″, or C-16″ of the aromatic moieties, given the molecular formula C_34_H_26_O_10_.

**Figure 3:**
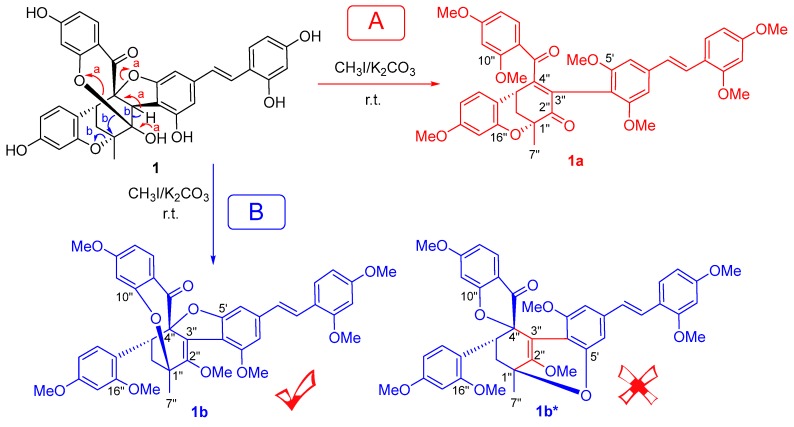
Methylation of compound **1.**

Because all the oxygenated bridgeheads (C-1″, 2″, 4″) were quaternary carbons, the methylation of compound **1** with CH_3_I/K_2_CO_3_ was carried out to confirm the linkages of three *O*-bridges. Two products—**1a** and **1b** as shown in Figure 3—were identified by the 1D and 2D-NMR spectra.

In **1a**, the ^13^C-NMR spectrum showed two carbonyl carbons at *δ*_C_ 193.6 (C-2″) and 193.0 (C-8″), two olefinic carbons at *δ*_C_ 127.7 (C-3″) and 160.4 (C-4″), four aliphatic carbons at *δ*_C_ 75.9 (C-1″), 33.7(C-5″), 35.6 (C-6″), and 22.7 (C-7″), and the aromatic moieties. The ^1^H-NMR spectrum showed one methyl at *δ*_H_ 1.57 (s, H-7″), one methine at *δ*_H_ 3.84 (t, *J* = 3.0 Hz, H-5″), and one methylene at *δ*_H_ 2.65, 2.23 (each dd, *J* = 13.3, 3.0 Hz, H-6″). The HMBC showed correlations from H-5″ to C-1″/C-3″, from H-6″ to C-1″/C-2″/C-5″, and from H-7″ to C-1″/C-2″/C-6″, establishing that the D-A skeleton was 1″-oxymethylcyclohex-3″-en-2″-one. In addition, the cross-peaks from H-2′ (*δ*_H_ 6.42) to C-3′ (*δ*_C_ 157.7) and from H-6′ (*δ*_H_ 6.41) to C-5′ (*δ*_C_ 157.8) provided the assignments for C-3′ and C-5′ of the stilbene. The cross-peaks from H-11″ (*δ*_H_ 6.39)/H-14″ (*δ*_H_ 7.04) to C-10″ (*δ*_C_ 159.7) and from H-11″/H-13″ (*δ*_H_ 6.33)/H-14″ (*δ*_H_ 7.04) to C-12″ (*δ*_C_ 164.2) provided the assignments for C-10″ and C-12″ of the benzoyl group. The cross-peaks from H-17″ (*δ*_H_ 6.47)/H-20″ (*δ*_H_ 6.97) to C-16″ (*δ*_C_ 153.8)/C-18″ (*δ*_C_ 160.2) provided the assignments of the oxygenated carbons (C-16″ and C-18″) of trisubstituted benzene. All of the methoxylated carbons were assigned by the cross-peaks from methyl groups to their *ipso* carbons, and only C-1″ and C-16″ were not substituted by a methyl group. Therefore, one *O*-bridge was assigned between C-1″ and C-16″.

In **1b**, the A-D skeleton was determined to be 1″,2″,4″-trioxymethylcyclohexene, which was deduced from one methyl at *δ*_H_ 1.57 (s, H-7″), one methylene at *δ*_H_ 2.28 (dd, *J* = 13.5, 1.2 Hz, H-6″*a*) and 1.87 (dd, *J* = 13.5, 4.2 Hz, H-6″*e*), and seven carbons at *δ*_C_ 76.4 (C-1″), 148.0 (C-2″), 122.4 (C-3″), 99.4 (C-4″), 33.9 (C-5″), 31.3 (C-6″), and 23.2 (C-7″). This was further confirmed by the HMBC correlations from the H-6″ to C-1″/C-2″/C-4″/C-5″ and from H-7″ to C-1″/C-2″/C-6″. In addition, the cross-peaks from H-2′ (*δ*_H_ 6.56) to C-3′ (*δ*_C_ 155.7) and from H-6′ (*δ*_H_ 6.36) to C-5′ (*δ*_C_ 161.8) provided the assignments for C-3′ and C-5′ of the stilbene moiety. The cross-peaks from H-11″ (*δ*_H_ 6.62)/H-14″ (*δ*_H_ 7.24) to C-10″ (*δ*_C_ 159.6)/C-12″ (*δ*_C_ 163.6) were used to assign C-10″ and C-12″ of the benzoyl group. The cross-peaks from H-17″ (*δ*_H_ 6.27)/H-20″ (*δ*_H_ 7.12) to C-16″ (*δ*_C_ 154.9)/C-18″ (*δ*_C_ 160.0) provided the assignments for the oxygenated aromatic carbon C-16″ and C-18″. All methoxylated carbons were assigned by the cross-peaks from methyl groups to their *ipso* carbons. Four carbons C-1″, C-4″, C-5′, and C-10″ were not substituted by a methyl group. Given the molecular formula C_41_H_4__0_O_10_ as calculated by HRMS, there should be two *O*-bridges in **1b** between C-1″/C-10″ and C-4″/C-5′, or between C-1″/C-5′ and C-4″/C-10″. Finally, the *O*-bridges were attributed at C-1″/C-10″ and C-4″/C-5′ due to the weak NOESY cross-peak between H-6″*a* (*δ*_H_ 2.28)/H-14″ (*δ*_H_ 7.24). The structure of **1b** could be further confirmed by the unreasonably twisted double bond C2″-C3″ that would be present if the *O*-bridges were located on C-1″/C-5′ and C-4″/C-10″ (1b* in Figure 3).

Given the structures of **1a** and **1b**, the three *O*-bridges in **1** were suggested to be at C-1″/C-16″, C-2″/C-10″, and C-4″/C-5′, depending on the proposed reaction mechanism. In the methylation of **1**, there were two reactive centers: the hemiketal at C-2″ and its adjacent benzyl proton H-3″. In pathway A, deprotonation at C-3″ under alkali conditions formed a ketone from the hemiketal. Subsequently, the two *O*-bridges at C-2″ and C-4″ were broken to form a 1,4-butenedione. In pathway B, the hydroxyl group at C-2″ hemiketal was first methylated before deprotonation at C-3″ under alkali conditions. A double bond was formed as the *O*-bridge at C-2″ migrated to C-1″ with an intramolecular 1,2-rearrangement, and the *O*-bridge between C-1″/C-16″ was broken. Meanwhile, a configuration inversion of the C-7″ methyl group was observed from **1** to **1b**. This phenomenon was further confirmation of the intramolecular *O*-bridge migration from C-2″ to C-1″.

**Figure 4:**
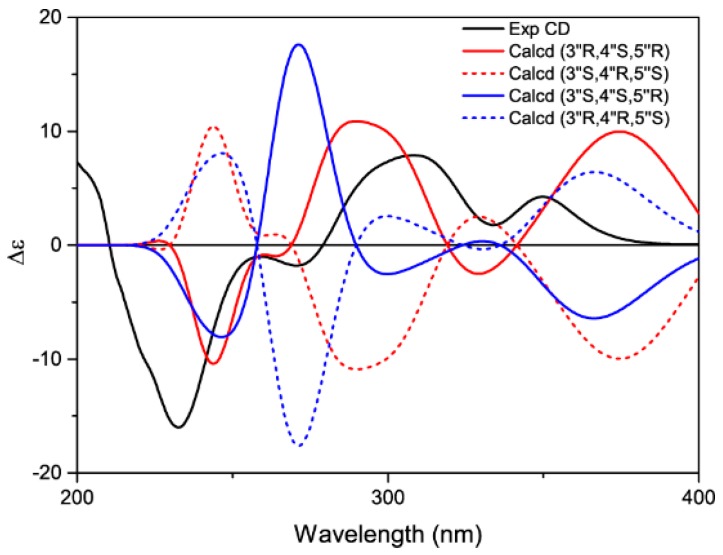
Experimental and calculated ECD (Electronic Circular Dichroism) of compound **1**.

Because the two bridged rings on C1″/C5″ and C2″/C4″ were adjacent to each other, they must be on opposite sides of the hexane plane. Thus, the orientation of C-3″ yielded two sets of epimers—*cis-trans* or *all-trans*, in agreement with the biosynthesis pathway [17] of the D-A adducts in the genus *Morus*. Although the benzyl carbonyl of **1** was coplanar with the aromatic ring, its CD (Circular Dichroism) spectrum did not show the split Cotton effects typical of non-*O*-bridged D-A adducts, such as mulberrofurans C and J [16]. Two positive Cotton effect peaks at 349 nm and 308 nm were observed, in accordance with the calculated ECD (Electronic Circular Dichroism) spectrum (Figure 4) of one 3″H-α epimer—i.e., (3″*R*, 4″*S*, 5″*R*)—by using the TDDFT (Time-Dependent Density Functional Theory) method. Therefore, the absolute configuration of **1** was determined to be (1″*R*, 2″*R*, 3″*R*, 4″*S*, 5″*R*).

**Figure 5:**
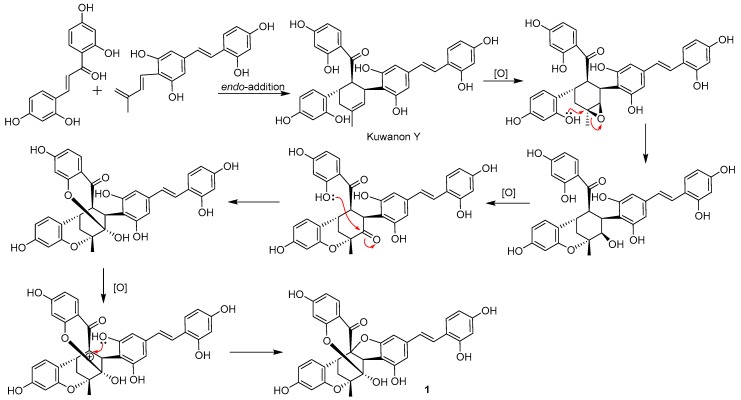
Plausible biosynthetic pathway of compound **1**.

The genus *Morus* is a plant source with rich D-A adducts. More than 50 D-A adducts have been found in the previous studies [18]. However, a natural product with a highly oxygenated D-A skeleton is rarely reported [19]. A plausible biosynthetic pathway for **1** was postulated in Figure 5, based on the KEGG pathway prediction. Kuwanon Y, a D-A adduct found in genus *Morus* [16], afforded **1** through three oxidization steps. First, the double bond of the D-A skeleton was oxidized to an epoxide by an oxidase [20] or putative Cyt P450 monooxygenase [19], then the epoxide was attacked by 16″-OH at C-1″, and 2″-OH was formed. Sequentially, the newly formed 2″-OH was oxidized to a carbonyl by an oxidoreductase [21] and was attracted by 10″-OH to form a hemiketal [22]. Finally, the α-position of the 8″-carbonyl was oxidized to form an electrophilic center and was trapped by 5′-OH [23] to afford **1**.

In in vitro bioassays, sanggenon X (**1**) showed significant antioxidant activity against Fe^2+^-Cys-induced lipid peroxidation in rat liver microsomes with 81.25% inhibition of malondialdehyde (MDA) release, similar to the positive control, curcumin, with an 81.75% inhibition ratio.

## 3. Experimental

### 3.1. General Experimental Procedures

Melting points were determined on an XT5B melting point apparatus (Beijing Keyi Electric Light Instrument Factory, Beijing, China) and were uncorrected. Optical rotations were measured with a P-2000 polarimeter (Jasco, Tokyo, Japan). ECD spectra were recorded at room temperature with a J-815 spectropolarimeter (Jasco, Tokyo, Japan). UV spectra were collected in MeOH on a V-650 spectrophotometer (Jasco, Tokyo, Japan). IR spectra were recorded on a Nicolet 5700 spectrometer (Thermo, Madison, WI, USA) by the FT-IR transmission electron microscopy method. ^1^H- and ^13^C-NMR spectra were acquired using an AVIIIHD 600 spectrometer (Bruker, Billerica, MA, USA). ESI HR-MS were recorded on a 1200 series LC/6520 quadrupole time of flight (QTOF) spectrometer (Agilent). Column chromatography (CC) purification was performed using silica gel (160–200 mesh), Sephadex LH-20 (GE, Boston, MA, USA), and C_18_ (50 μm, YMC, Kyoto, Japan). CC fractions were analyzed by thin-layer chromatography (TLC) using silica gel GF_254_.

### 3.2. Plant Material

The Cortex Mori Radicis were bought from Anguo herb market, Hebei, China, and were collected from Hunan Province, China, in 2012. These samples were identified by Professor Lin Ma, Institute of Materia Medica, Chinese Academy of Medical Science and Peking Union Medical College, China. A voucher specimen (ID-S-2604) was deposited in the Institute of Materia Medica, Chinese Academy of Medical Science and Peking Union Medical College, China.

### 3.3. Extraction and Isolation

The powdered Cortex Mori Radicis (50 kg) were soaked with 50% EtOH for 24 h and percolated with 300 L 50% EtOH. Then evaporation of the solvent under reduced pressure gave a liquid extract, which was suspended in H_2_O and partitioned with EtOAc. The EtOAc extract (ca. 1 kg) was applied to a silica gel (100–200 mesh, 2 kg) column, eluting with a gradient of increasing MeOH concentration (0–100%) in CHCl_3_, to yield 22 fractions A–V. Fraction M–O (50 g) was applied to a Sephadex LH-20 (3 L) column, using 90% MeOH as eluent, to give subfractions MO-1 to 13. Fraction MO-11 (8 g) was loaded on a silica gel (100–200 mesh, 160 g) column and eluted with a gradient of increasing MeOH concentration (0–100%) in CH_2_Cl_2_ to yield five subfractions. The second fraction (3.2 g) was chromatographed over Sephadex LH-20 (400 mL, eluted by MeOH), MPLC over C_18_ (eluted by MeOH:H_2_O 10–60%), and HPLC (YMC C_18_ 20 × 250 mm, 5 μm, 65% MeOH in H_2_O, flow rate 5 mL/min) to give **1** (68 mg, *t*_R_ = 39 min).

Sanggenon X (**1**): Yellowish-brown amorphous powder; m.p. 199.0–200.3 °C (d); ${\left[\alpha \right]}_{D}^{20}$ = −8.76° (*c* = 1.00, MeOH); UV (MeOH) λ_max_ (log*ε*) 208.5 (4.73), 285 (4.40), 326 (4.51) nm; CD (MeOH) 232.5 (∆*ε* −16.00), 308 (∆*ε* +7.88), 349.5 (∆*ε* +4.24) nm; IR υ_max_ 3392, 1685, 1605, 1509, 1459, 1279, 1217, 1165, 1125, 1064, 995, 973, 838, 767, 661, 636, 525 cm^−1^; ^1^H-NMR (DMSO-*d*_6_, 600 MHz) 9.55 (1H, s 2-OH), 9.38 (1H, s 4-OH), 8.85 (1H, s 3′-OH), 6.64 (1H, s 2″-OH), 9.29 (1H, s 18″-OH), other data see Table 1; ^13^C-NMR (DMSO-*d*_6_, 150 MHz) data, see Table 1; (+)-ESIMS *m*/*z* 595 [M + H]^+^, 617 [M + Na]^+^; (+)HR-ESIMS *m*/*z* 595.1586 [M + H]^+^ (calcd. for C_34_H_27_O_10_^+^, 595.1599). (Supplementary Materials Figure S1a–j, Table S1).

**1a**: (+)HR-ESIMS *m*/*z* 693.2708 [M + H]^+^ (calcd for C_41_H_41_O_10_^+^, 693.2694). ^1^H-NMR (DMSO-*d*_6_, 600 MHz) 3.44 s (2-OMe), 3.77 (3H, s, 4-OMe), 3.37 (3H, s, 3′-OMe), 3.82 (3H, s, 5′-OMe), 3.71 3H, s, 10″-OMe), 3.71 (3H, s, 12″-OMe), 3.71 (3H, s, 18″-OMe), other data see Table 1. ^13^C-NMR (DMSO-*d*_6_, 150 MHz) 55.2 (2-OMe), 55.3 (4-OMe), 55.7 (3′-OMe), 55.6 (5′,10″,12″,18″-OMe), other data see Table 1. (Supplementary Materials Figure S2a–f, Table S1).

**1b**: (+)HR-ESIMS *m*/*z* 693.2708 [M + H]^+^ (calcd. for C_41_H_41_O_10_^+^, 693.2694). ^1^H-NMR (DMSO-*d*_6_, 600 MHz) 3.77 (3H, s, 2-OMe), 3.73 (3H, s, 4-OMe), 3.76 (9H, s, 3′,12″,16″-OMe), 3.32 (3H, s, 2″-OMe), 3.60 (3H, s, 18″-OMe), other data see Table 1. ^13^C-NMR (DMSO-*d*_6_, 150 MHz) 56.0 (2-OMe), 55.8 (4-OMe), 56.1 (3′,16″-OMe), 60.7 (2″-OMe), 56.2 (12″-OMe), 55.5 (18″-OMe), other data see Table 1. (Supplementary Materials Figure S3a–h, Table S1).

### 3.4. Methylation of ***1***

Twenty milligrams of **1** was dissolved in dried acetone, 200 mg K_2_CO_3_ and 400 μL CH_3_I were added and then stirred for 24 h. Then, the solution was dried and purified by RP-HPLC (Grace Adsorbosphere XL C_18_ 10 × 250 mm, 5 μm, 90% MeOH in H_2_O, flow rate 2 mL/min) to yield compounds **1a** and **1b** (**1a**: 3.5 mg, 11.7%, *t*_R_ = 11.9 min; **1b**: 3.4 mg, 11.3%, *t*_R_ = 25.9 min).

### 3.5. Calculation of ECD

Calculated ECD was performed on the 3″H-α (1″*R*, 2″*R*, 3″*R*, 4″*S*, 5″*R*), 3″H-β (1″*R*, 2″*R*, 3″*S*, 4″*S*, 5″*R*), and their enantiomers of **1**. Conformation search was done with the MMFF94 molecular mechanics force field via the MOE software package (MOE2009.10, Chemical Computing Group Inc., Montreal, QC, Canada). Calculated ECD was performed using the TDDFT method (Gaussian 09 B.01, Gaussian, Wallingford, CT, USA, 2009) at B3LYP/6-31+G(d,p)//B3LYP/6-311+G(d,p) level for the configurations within an energy window of 5 kcal/mol. The conductor-like polarizable continuum model was used with MeOH (*ε* = 32.613) in order to take the solvent effects into consideration. The Boltzmann distribution was calculated based on the relative free energy (ΔG) and the final ECD (*σ* = 0.25 eV, UV shift = 10 nm) was simulated by using SpecDis (V1.64, University of Wuerzburg, Germany, 2015).

### 3.6. Lipid Peroxidation Assay

Antioxidative activity was evaluated as the inhibitory activity of compounds against Fe^2+^-Cys-induced lipid peroxidation in rat liver microsomes by the formation of malondialdehyde-thiobarbituric acid (MDA-TBA) adduct. Microsomes were isolated from SD rat livers and suspended in 100 mM TMS buffer (pH 7.4). The microsomal suspension (1 mg protein/mL), different concentrations of compound or vehicle, and 0.2 mM cysteine in 0.1 M PBS (pH 7.4) were incubated at 37 °C for 15 min, 50 µM FeSO_4_ was added, and the reaction mixture was then incubated at 37 °C for 15 min again. An equal volume of 20% (*w*/*v*) TCA (Trichloroacetic Acid) and 0.6% (*w*/*v*) TBA were added and kept in a boiling water bath for 10 min. After the mixture was centrifuged at 3000× *g* for 10 min, the absorbance of supernatant was measured at 532 nm and the concentration of MDA was calculated as C = (OD − 0.006)/0.07 × 10 nmol/mL. Lipid peroxidation inhibitory activity was calculated as follows: [1 − (T − B)/(C − B)] × 100%, in which T, C, and B are MDA concentrations of the sample treated, the control without sample, and the zero time control, respectively. Curcumin (10^−4^ M) was used as the positive control.

## 4. Conclusions

In this paper, a tri-*O*-bridged D-A adduct, sanggenon X (**1**), was isolated from a 55% alcohol extract of Cortex Mori Radicis. Given its complex structure with several quaternary carbons in the bridgeheads, it was fortunate for us to determine the exact structure with the help of chemical methylation and calculated ECD. The structure of **1**, with highly oxygenated D-A skeleton, adds a new skeletal entity to the natural D-A adducts and provides a new framework for synthesis and biological evaluation in the future. 

## Figures and Tables

**Table 1:** NMR spectroscopic data for compounds **1**, **1a**, and **1b** in DMSO-*d*_6_ (*J* in Hz).

Position	1		1a		1b	
	*δ*_H_	*δ*_C_^†^	*δ*_H_	*δ*_C_^‡^	*δ*_H_	*δ*_C_^†^
1		115.2		118.2		118.4
2		156.0		156.8		158.4
3	6.30 s	102.5	6.57 d (2.2)	98.4	6.51 s ^c^	98.7
4		158.1		160.4		161.1
5	6.23 d (8.4) ^a^	107.1	6.54 dd (8.8, 2.2)	105.6	6.50 d (8.4) ^c^	106.2
6	7.30 d (8.4)	127.2	7.49 d (8.8)	127.6	7.45 d (8.4)	128.3
α	7.09 d (16.2)	123.1	7.16 d (16.5)	123.1	7.15 d (16.8)	124.6
β	6.74 d (16.2)	124.7	6.91 d (16.5)	127.0	6.89 d (16.8)	126.6
1′		139.3		139.3		141.8
2′	6.16 s	105.7	6.42 s ^b^	101.1	6.56 s	104.5
3′		154.2		157.7		155.7
4′		110.7		110.7		110.7
5′		159.2		157.8		161.8
6′	6.44 s	98.8	6.41 s	101.7	6.36 s	100.1
1″		74.4		75.9		76.4
2″		109.1		193.6		148.0
3″	3.17 s	47.3		127.7		122.4
4″		91.4		160.4		99.4
5″	2.66 brs	36.6	3.84 t (3.0)	33.7	N.D.	33.9
6″	2.51 d (13.8) * 1.77 dd (13.8, 3.0)	30.1	2.65 dd (13.3, 3.0) 2.23 dd (13.3, 3.0)	35.6	2.28 dd (13.5, 1.2) 1.87 dd (13.5, 4.2)	31.3
7″	1.61 s	22.1	1.57 s	22.7	1.57 s	23.2
8″		194.9		193.0		202.1
9″		112.8		119.4		120.7
10″		166.2		159.7		159.6
11″	6.09 s	97.6	6.39 d (1.9)	97.5	6.62 d (2.1)	99.0
12″		171.6		164.2		163.6
13″	6.48 d (8.7)	111.4	6.33 dd (8.8, 1.9)	105.3	6.50 dd (8.7, 2.1) ^c^	105.8
14″	7.44 d (8.7)	125.7	7.04 d (8.8)	131.3	7.24 d (8.7)	131.5
15″		107.9		115.1		112.9
16″		154.6		153.8		154.9
17″	6.23 s ^a^	106.5	6.47 d (2.4)	101.4	6.27 d (2.4)	101.5
18″		157.7		160.2		160.0
19″	6.22 d (9.0) ^a^	101.9	6.43 dd (8.4, 2.4) ^b^	106.7	6.43 dd (8.4, 2.4)	106.9
20″	6.51 d (9.0)	133.3	6.97 d (8.4)	129.5	7.12 d (8.4)	133.0

^a–c^ The signals overlapped with each other. * Half of this signal was overlapped by a solvent peak. Measured at 150 MHz ^†^ or 125 MHz ^‡^ for ^13^C.

## Supplementary Materials

The supplementary materials are available online. Copies of MS, UV, ECD, IR, and NMR spectra of compounds **1**, **1a**, and **1b** are available online.
