# Supplementary material for: A Tri-O-Bridged Diels-Alder Adduct from Cortex Mori Radicis
Source: Molecules. 2018 Jan 9;23(1):133. doi: 10.3390/molecules23010133 (PMC6017575; doi:10.3390/molecules23010133)
Supplement: Supplementary file 1 [file molecules-23-00133-s001.pdf]

A tri-*O*-bridged Diels-Alder adduct from Cortex Mori Radicis

An-Qi Lu[1,†], Ming-Hua Chen[2,†], Jie Gao[3], Lu Wang[1], Han-Yu Yang[1], Lan Li[1], Bo Zhang[1], Hao-Ke He[1], Su-Juan Wang[1,*]

[1] State Key Laboratory of Bioactive Substance and Function of Natural Medicines, Institute of Materia Medica, Chinese Academy of Medical Sciences and Peking Union Medical College, Beijing 100050, China; anqilu@163.com (A.-Q. L.); mingsunlight@sina.com (M.-H. C.); wanglu@imm.ac.cn (L. W.); yanghanyu@imm.ac.cn (H.-Y. Y.); lilan92@outlook.com (L. L.); zhangbo@imm.ac.cn (B. Z.); hehaoke@imm.ac.cn (H.-K. H.)

[2] Institute of Medicinal of Biotechnology, Chinese Academy of Medical Sciences and Peking Union Medical College, Beijing 100050, China;

[3] GRU Cancer Center, Augusta University, Augusta, Georgia, 30912, United States; jgao@augusta.edu

* Correspondence: sujuanwangl@imm.ac.cn (S.-J. W.)

† These authors contributed equally to this paper.

Supporting informations

For Compound 1..S-3

Figure SI-1a (+)ESIMS spectra of compound **1**

Figure SI-1b (+)HR-ESIMS spectra of compound **1**

Figure SI-1c IR spectra of compound **1**

Figure SI-1d CD and UV spectrum of compound **1**

Figure SI-1e ^{1}HNMR spectra of compound **1**

Figure SI-1f ^{13}CNMR spectra of compound **1**

Figure SI-1g DEPT spectra of compound **1**

Figure SI-1h HSQC spectra of compound **1**

Figure SI-1i HMBC spectra of compound **1**

Figure SI-1j NOESY spectra of compound **1**

For Compound 1a..S-8

Figure SI-2a (+)ESIMS spectra of compound **1a**

Figure SI-2b (+)HR-ESIMS spectra of compound **1a**

Figure SI-2c ^{1}HNMR spectra of compound **1a**

Figure SI-2d ^{13}CNMR spectra of compound **1a**

Figure SI-2e HSQC spectra of compound **1a**

Figure SI-2f HMBC spectra of compound **1a**

For Compound 1b……………………………………………………………………………………………S11
Figure SI-3a (+)ESIMS spectra of compound **1b**
Figure SI-3b (+)HR-ESIMS spectra of compound **1b**
Figure SI-3c ^{1}HNMR spectra of compound **1b**
Figure SI-3d ^{13}CNMR spectra of compound **1b**
Figure SI-3e HSQC spectra of compound **1b**
Figure SI-3f HMBC spectra of compound **1b**
Figure SI-3g NOESY spectra of compound **1b**
Figure SI-3h "wrong" structure and the twisted bond of **1b***..................................S-15

Table SI-1 HMBC correlationships of compounds **1**, **1a** , and **1b** (H→C) ………………S-15

For Compound 1:

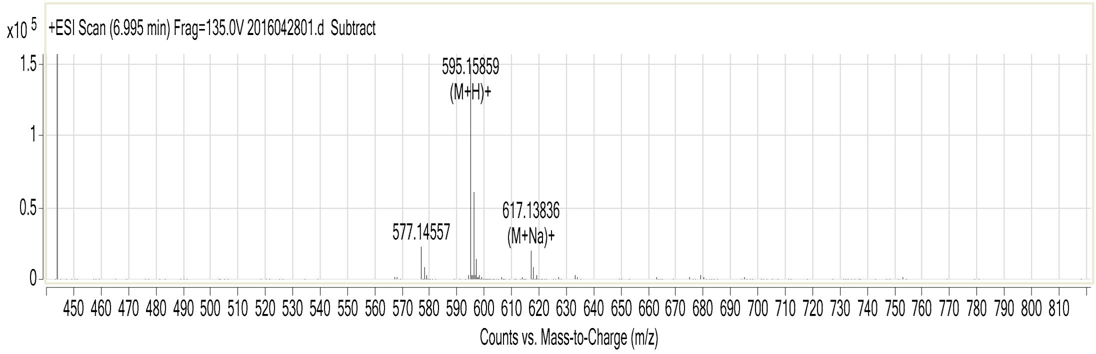

Figure SI-1a (+)ESIMS spectra of compound **1**

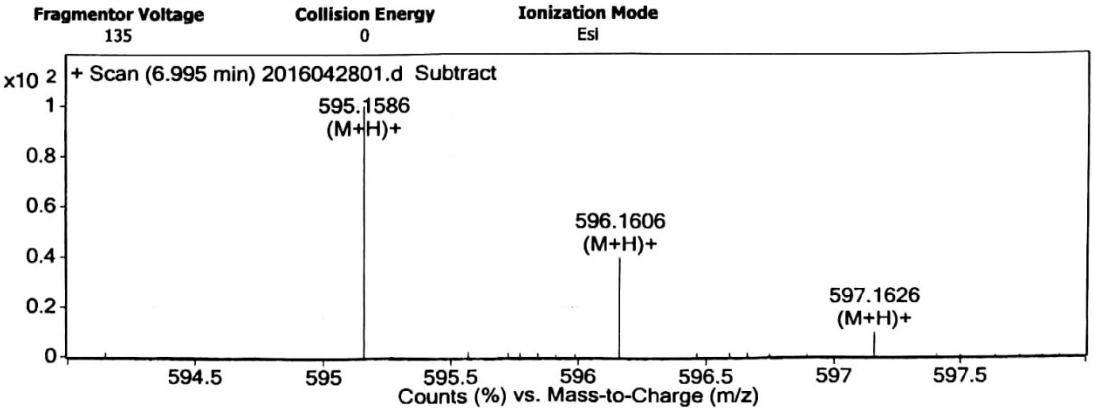

Figure SI-1b (+)HR-ESIMS spectra of compound **1**

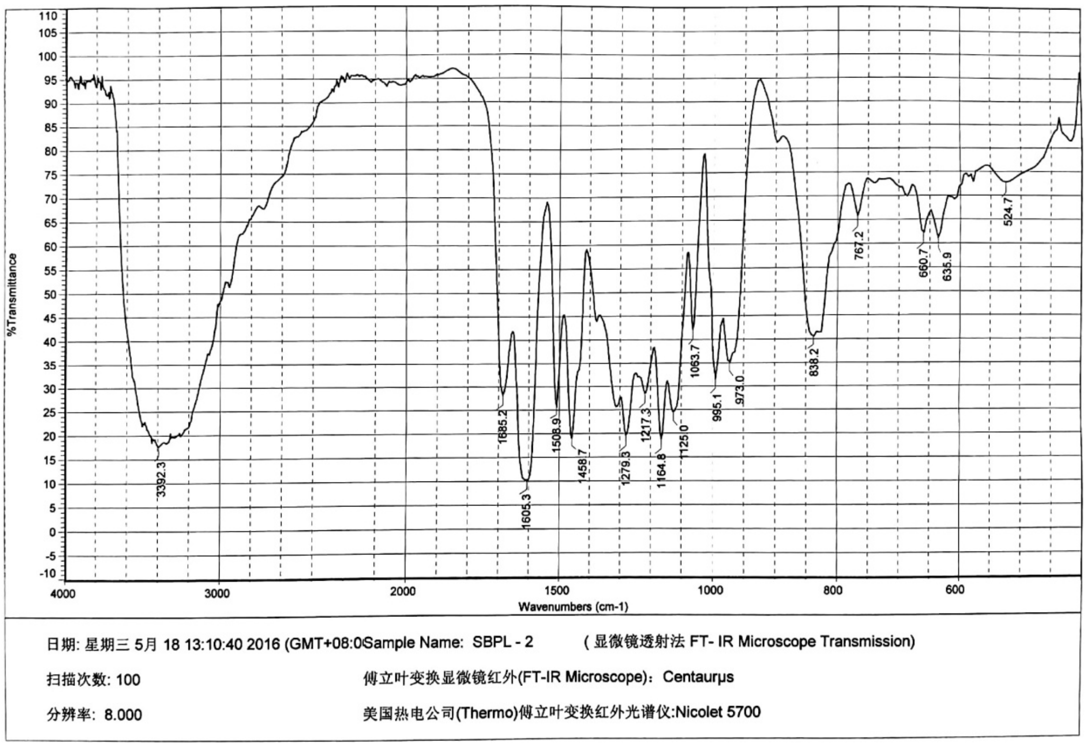

Figure SI-1c IR spectra of compound **1**

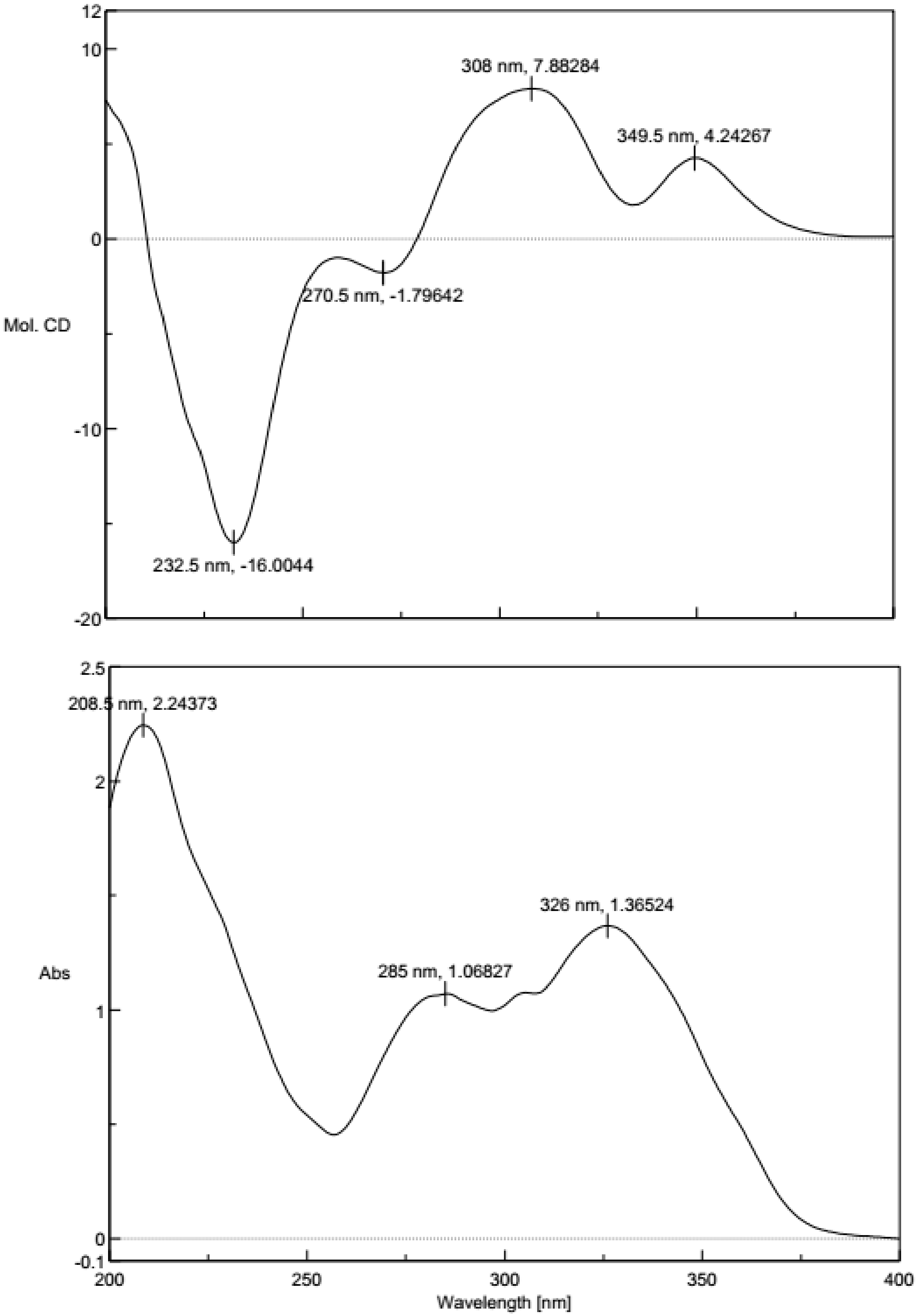

Figure SI-1d CD and UV spectrum of compound **1**

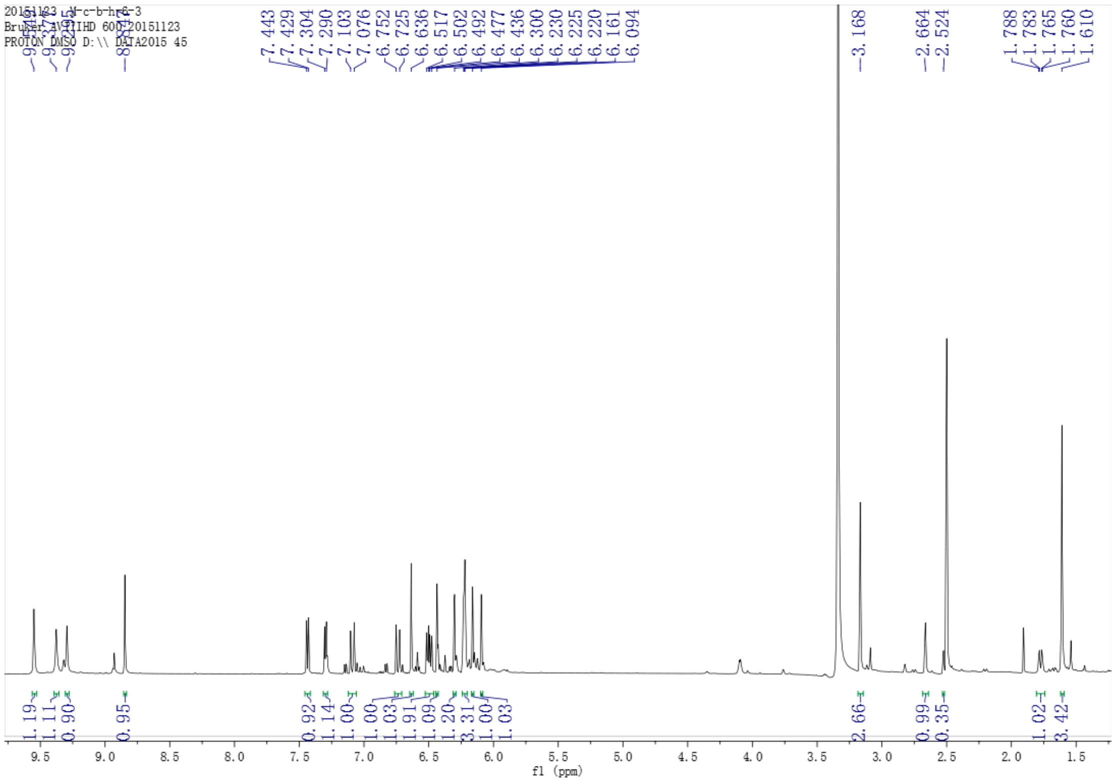

Figure SI-1e ^{1}HNMR spectra of compound **1**

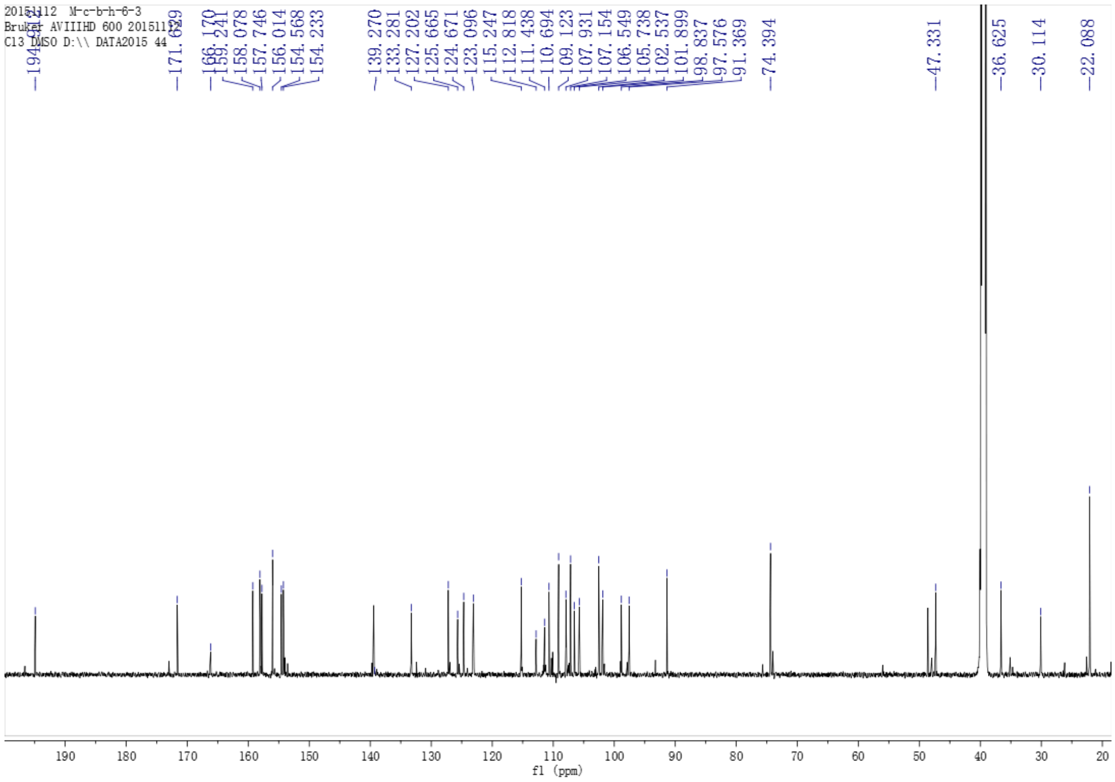

Figure SI-1f ^{13}CNMR spectra of compound **1**

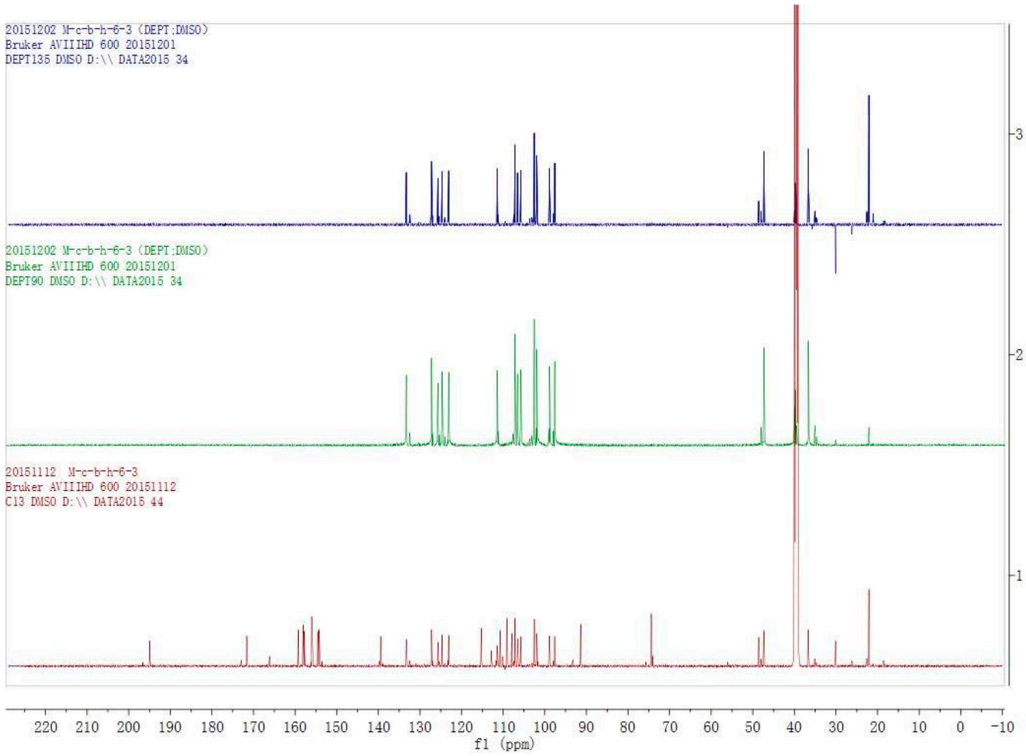

Figure SI-1g DEPT spectra of compound **1**

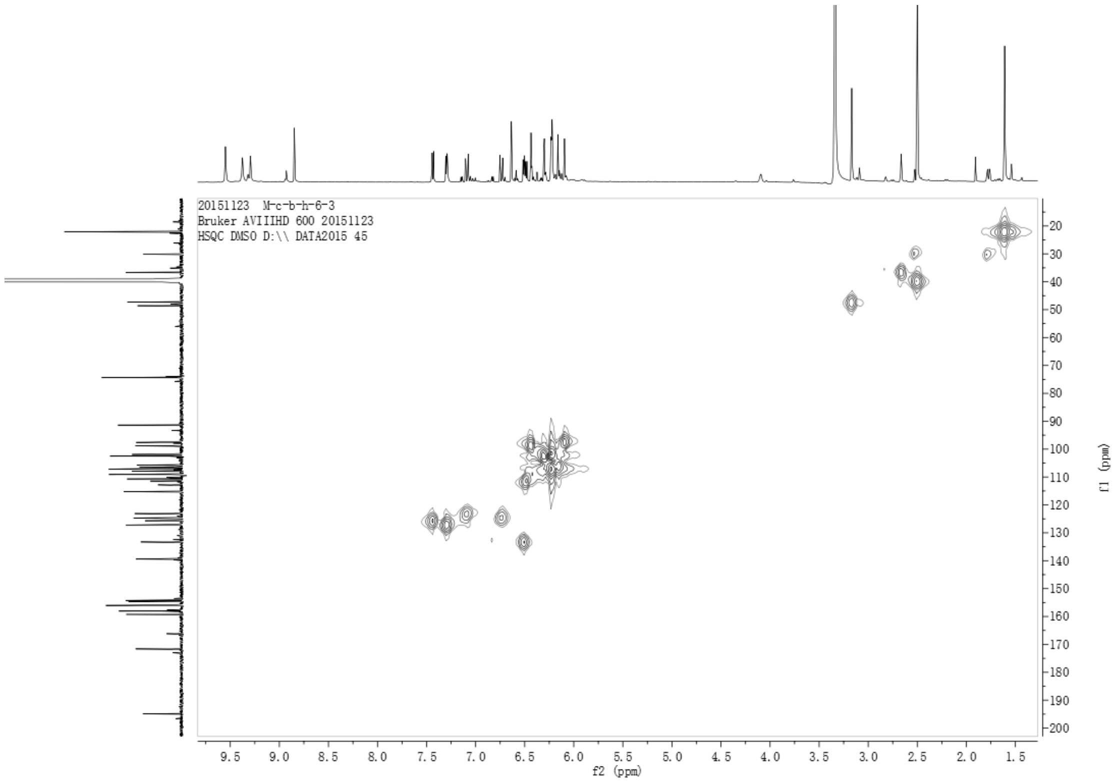

Figure SI-1h HSQC spectra of compound **1**

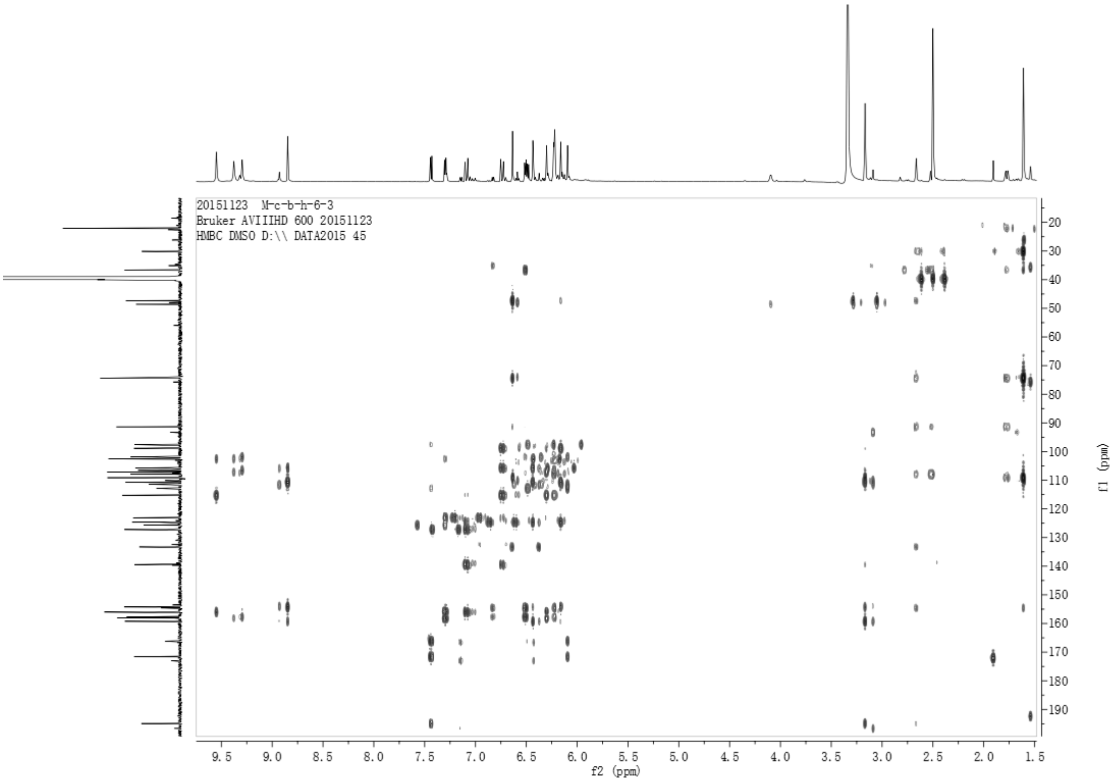

Figure SI-1i HMBC spectra of compound **1**

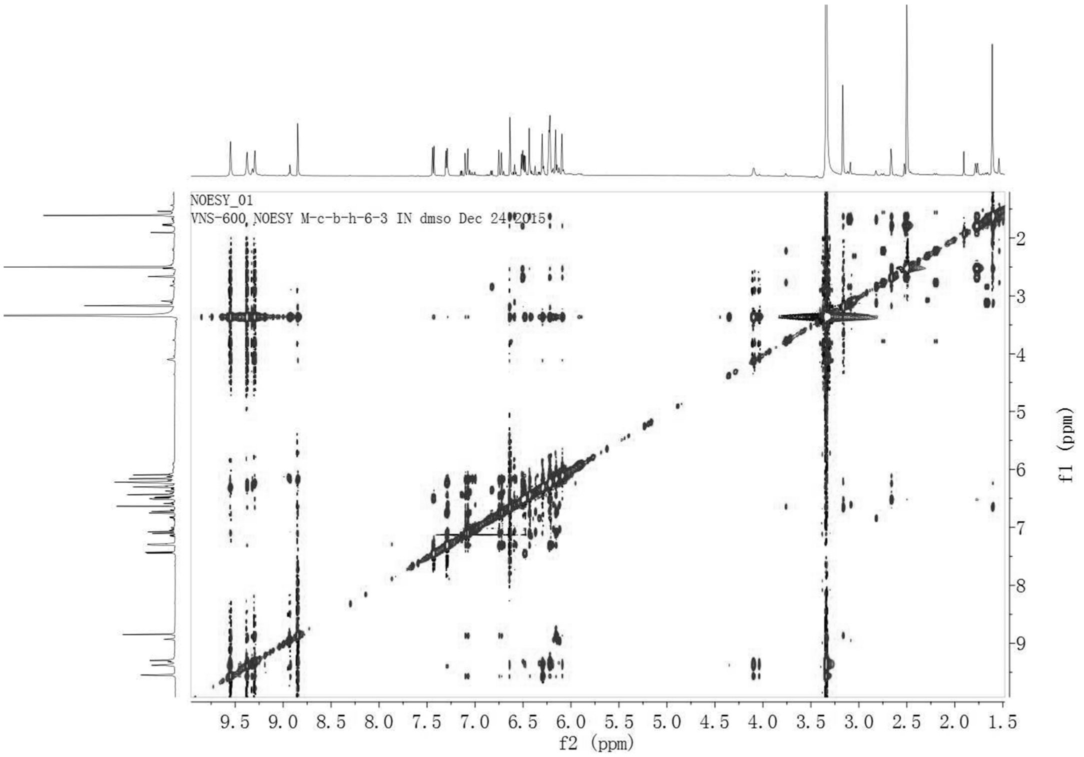

Figure SI-1j NOESY spectra of compound **1**

For Compound 1a:

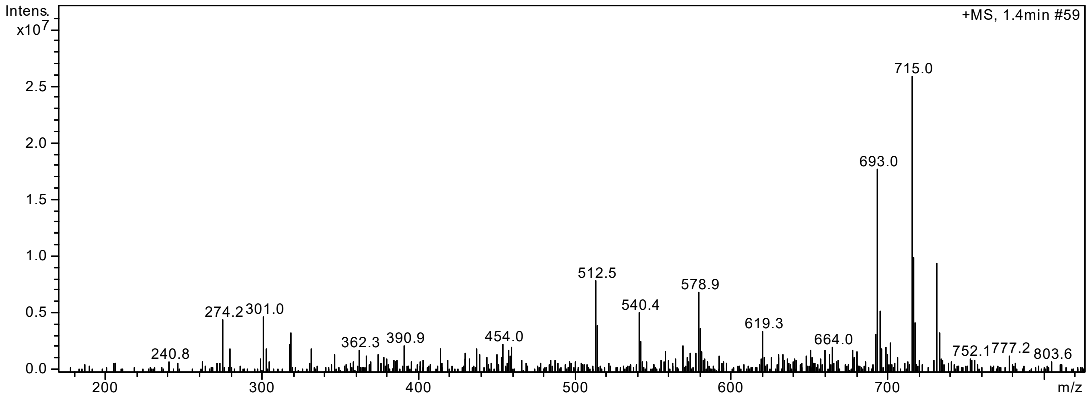

Figure SI-2a (+)ESIMS spectra of compound **1a**

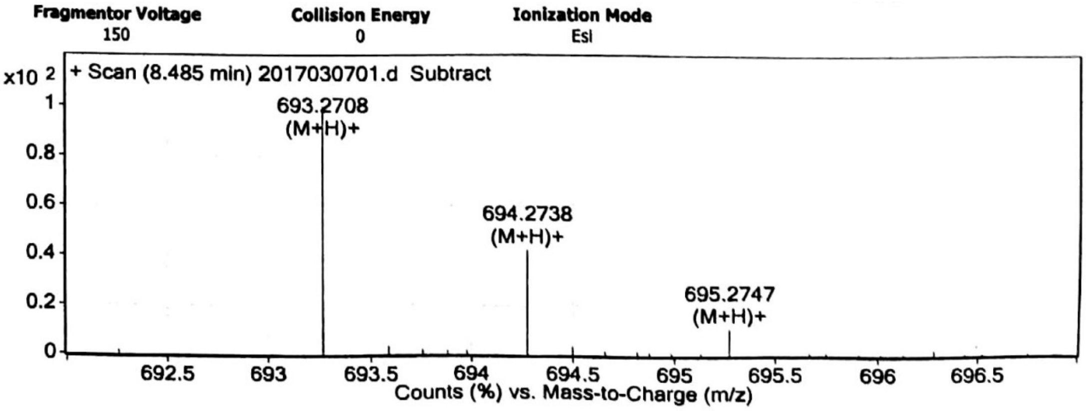

Figure SI-2b (+)HR-ESIMS spectra of compound **1a**

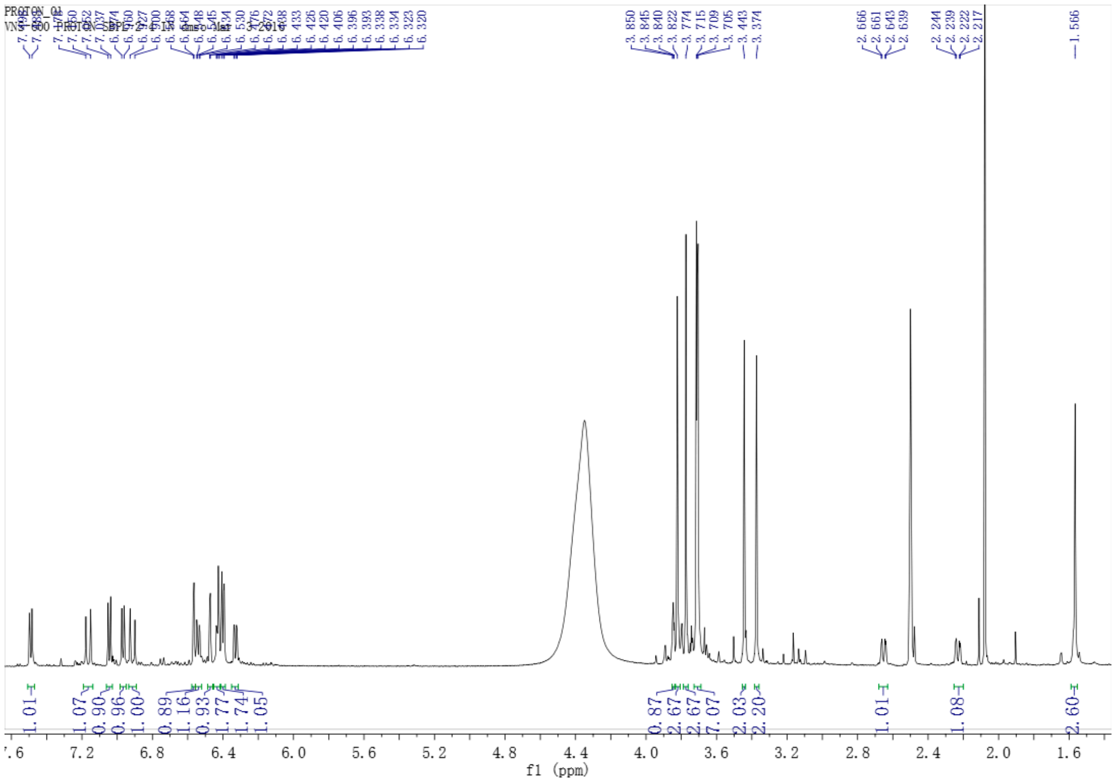

Figure SI-2c ^{1}HNMR spectra of compound **1a**

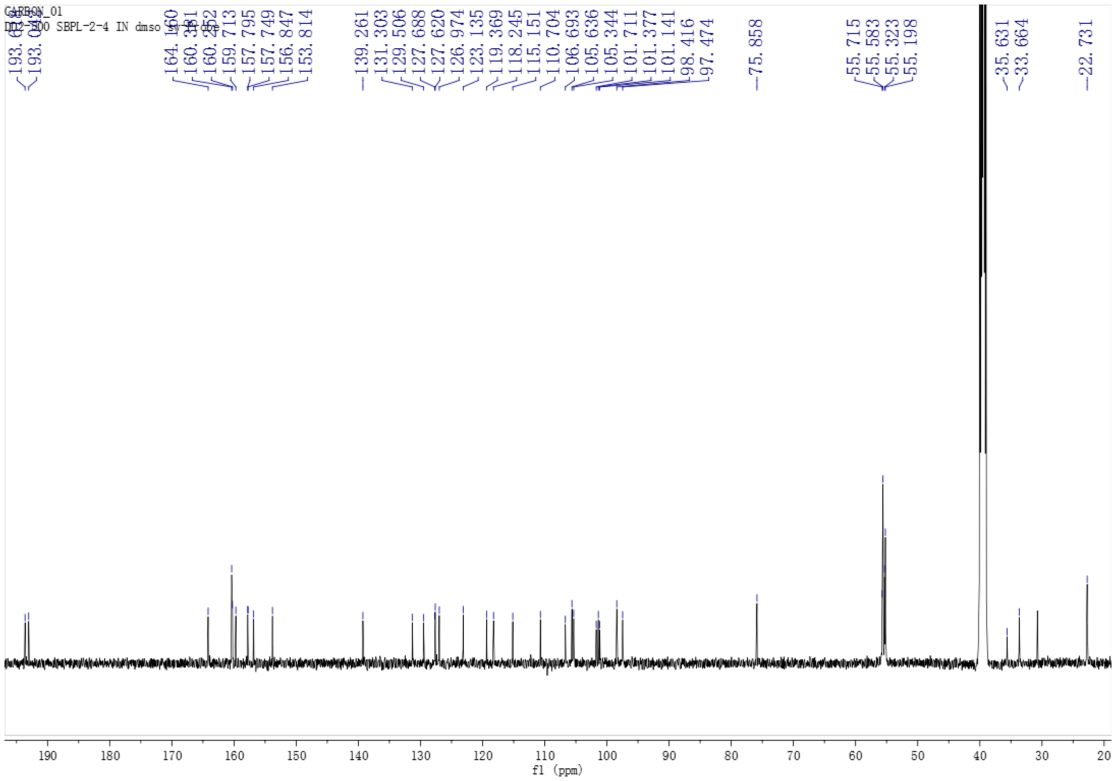

Figure SI-2d ^{13}CNMR spectra of compound **1a**

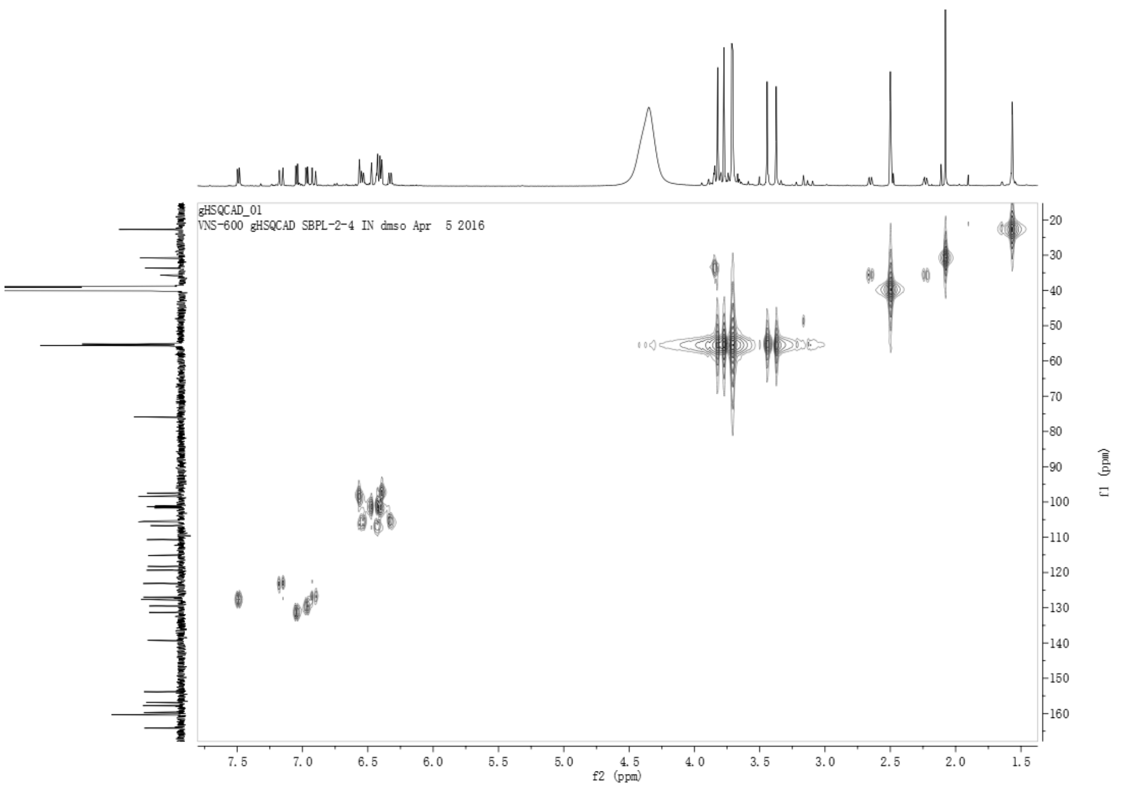

Figure SI-2e HSQC spectra of compound **1a**

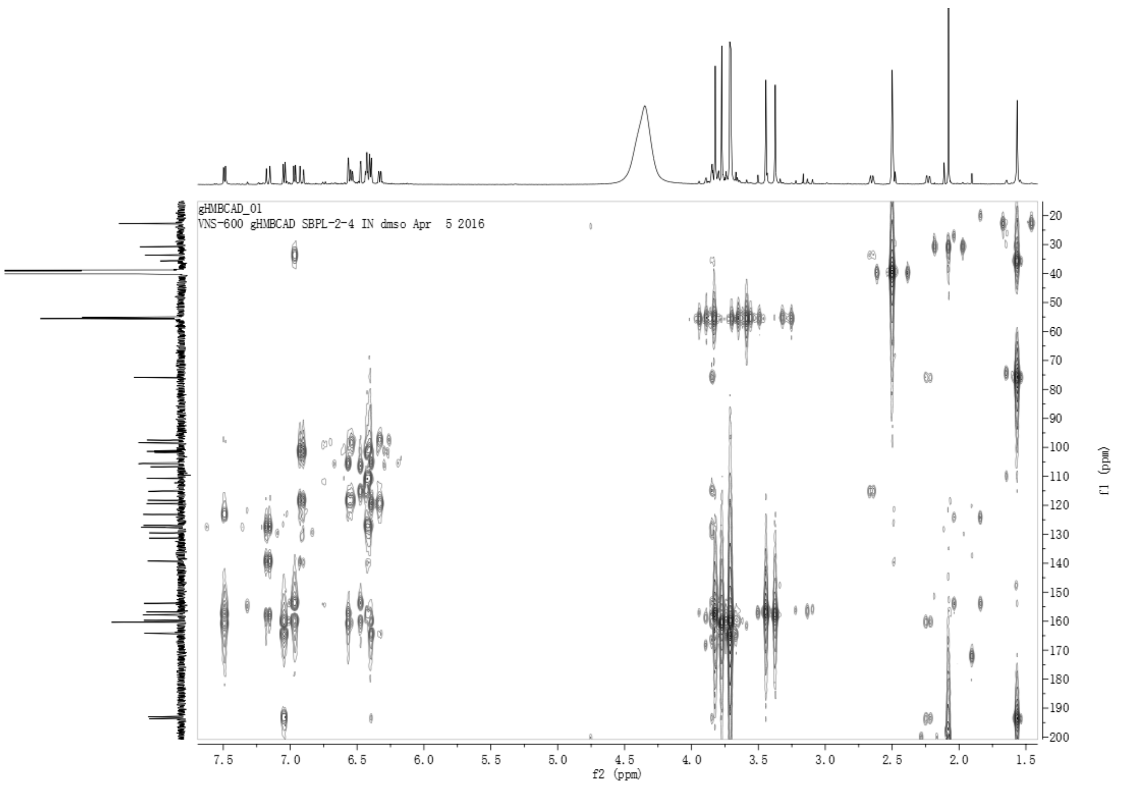

Figure SI-2f HMBC spectra of compound **1a**

For Compound 1b:

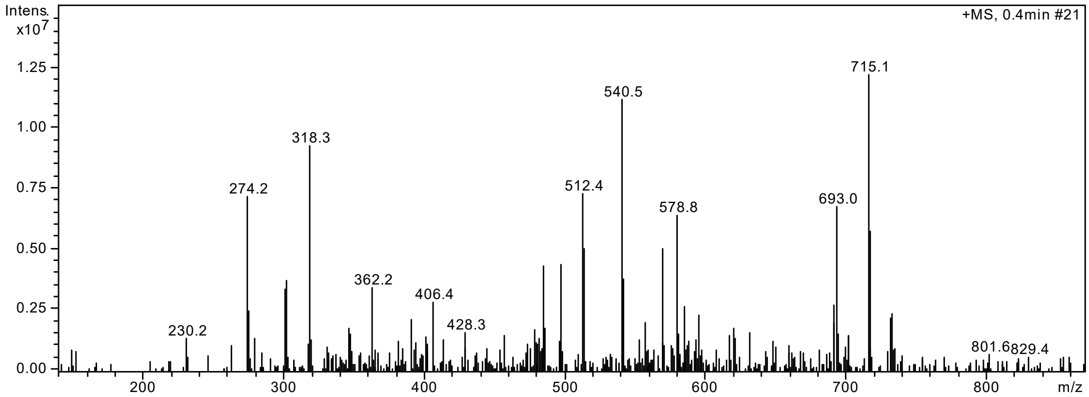

Figure SI-3a (+)ESIMS spectra of compound **1b**

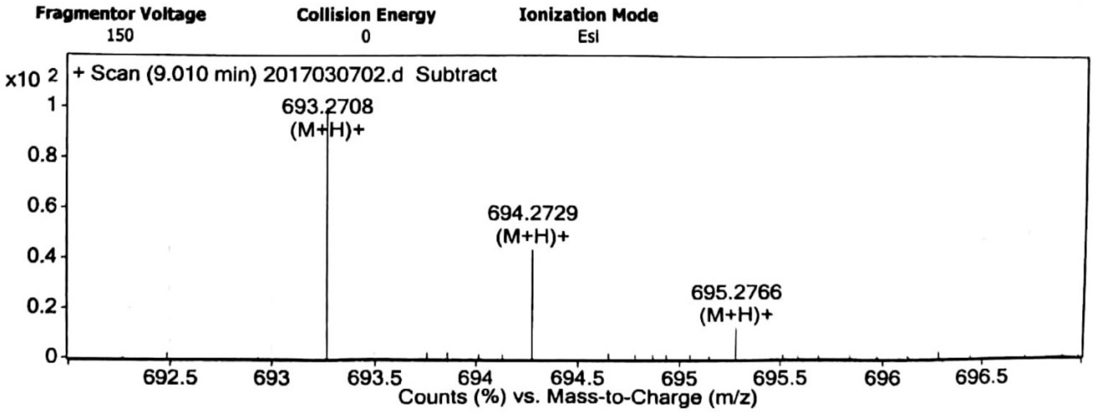

Figure SI-3b (+)HR-ESIMS spectra of compound **1b**

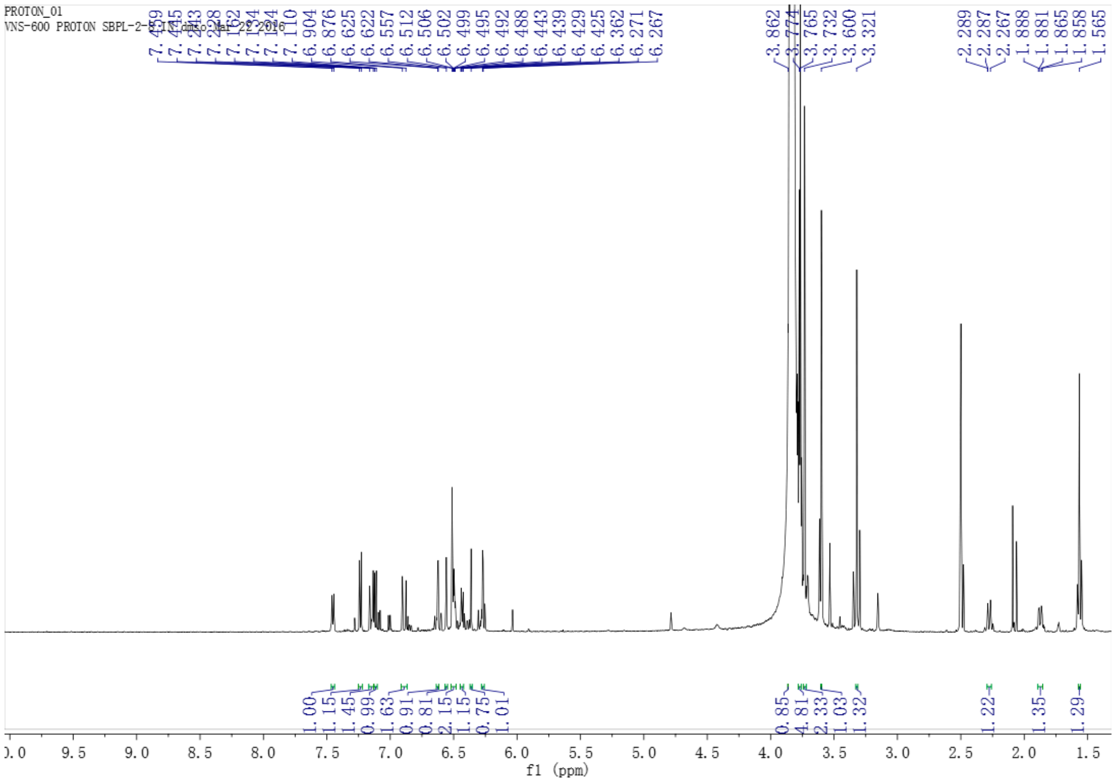

Figure SI-3c ^{1}HNMR spectra of compound **1b**

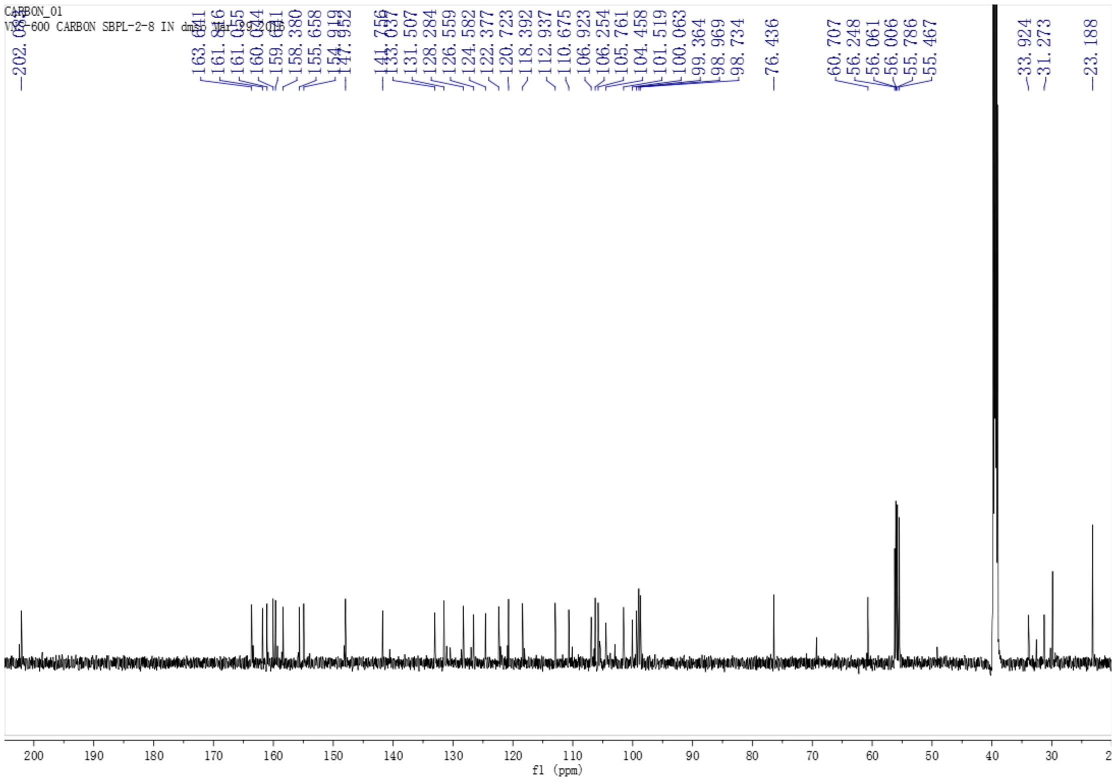

Figure SI-3d ^{13}CNMR spectra of compound **1b**

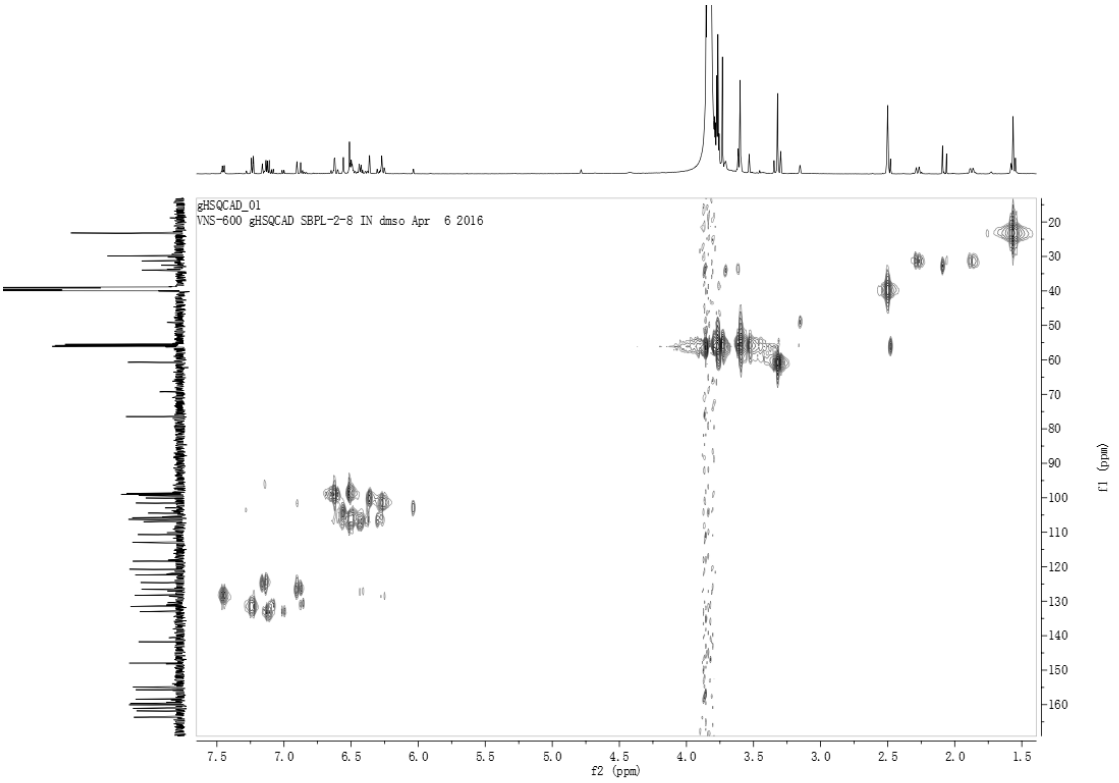

Figure SI-3e HSQC spectra of compound **1b**

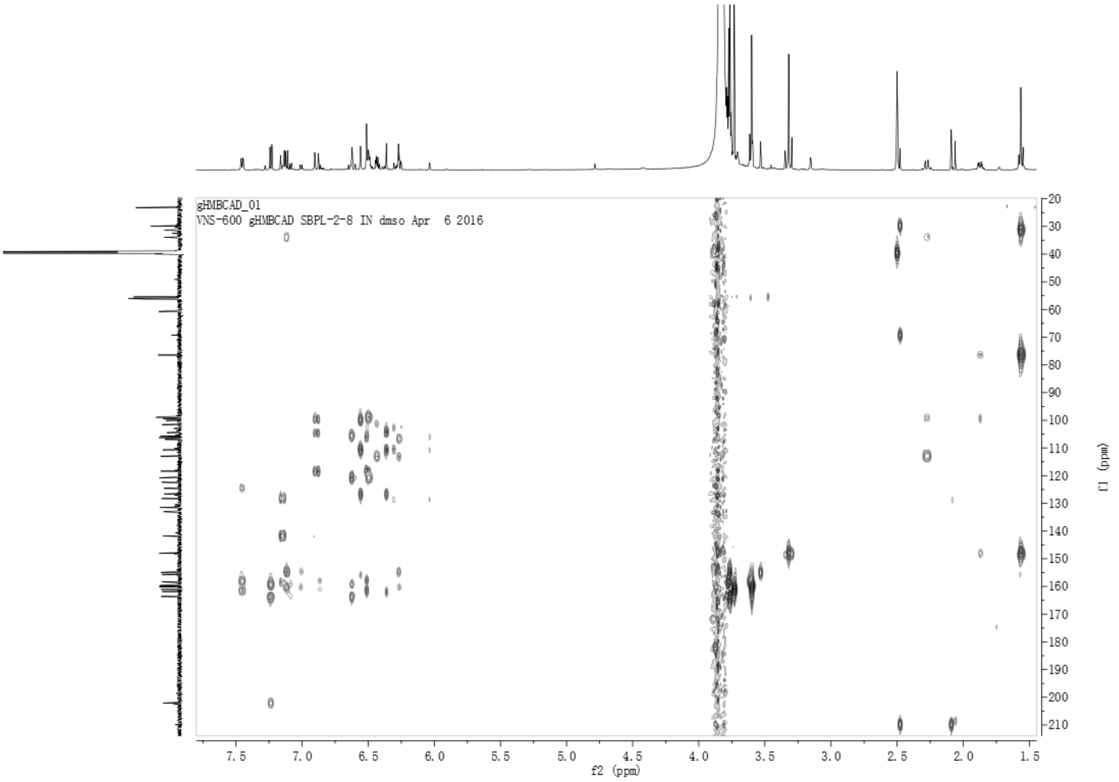

Figure SI-3f HMBC spectra of compound **1b**

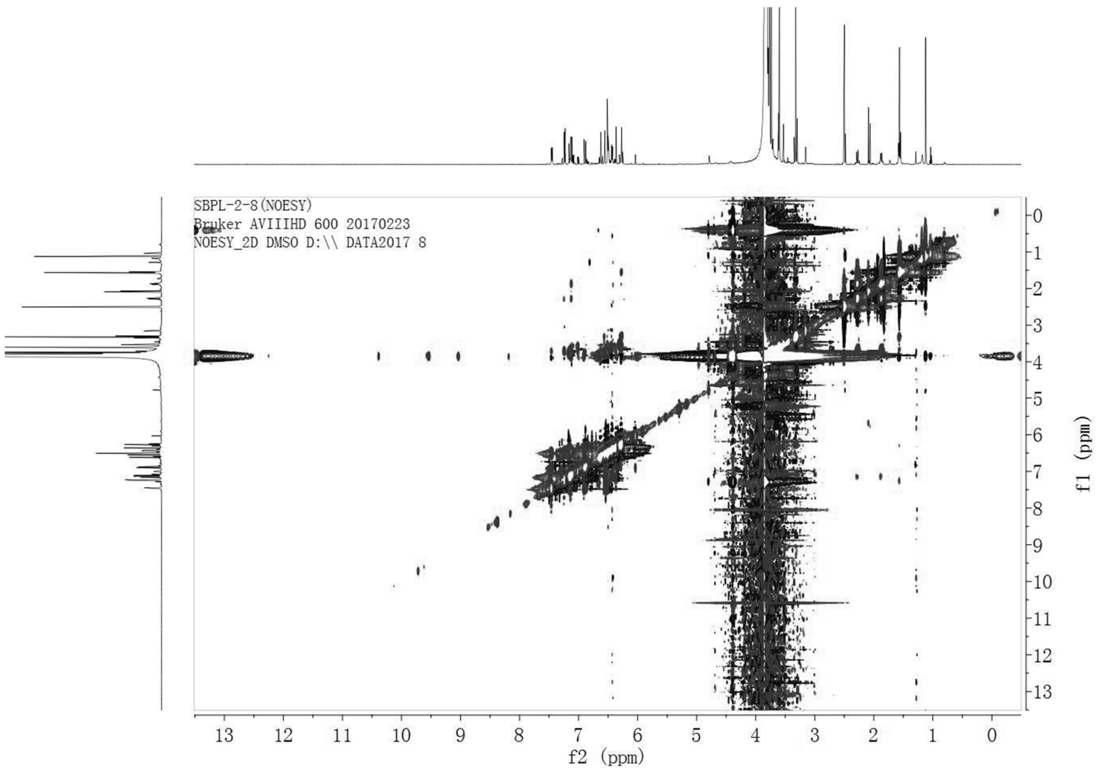

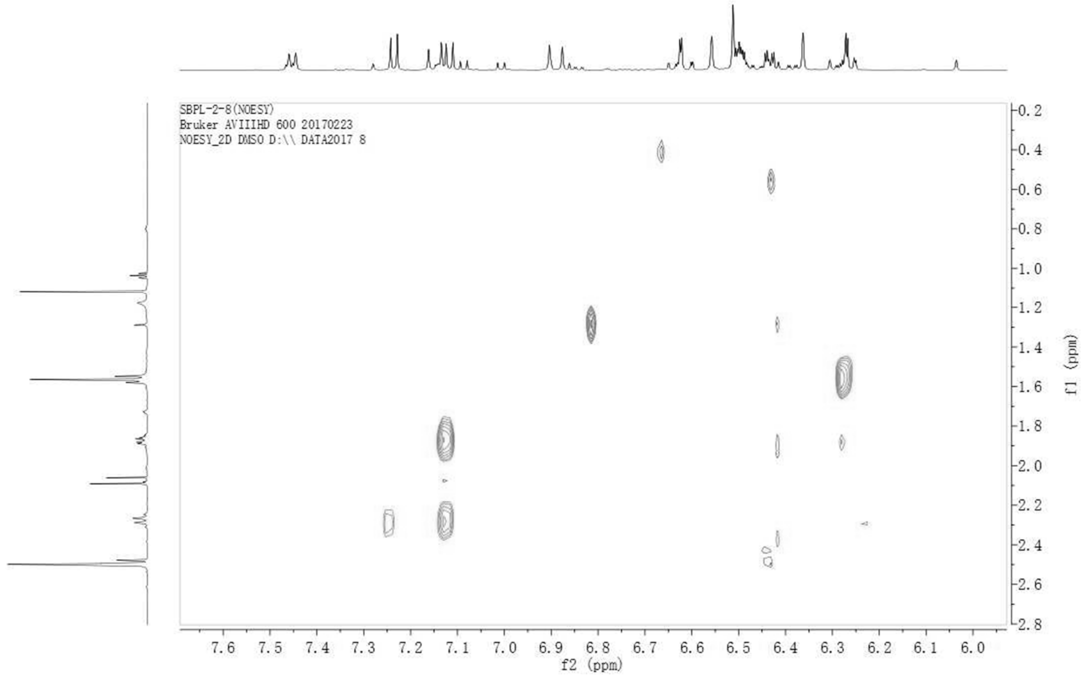

Figure SI-3g NOESY spectra of compound **1b**

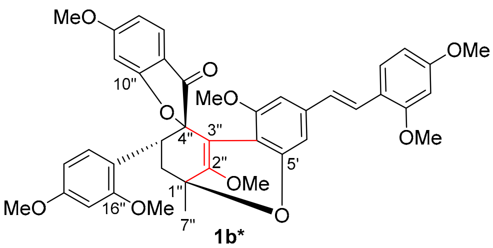

1b*

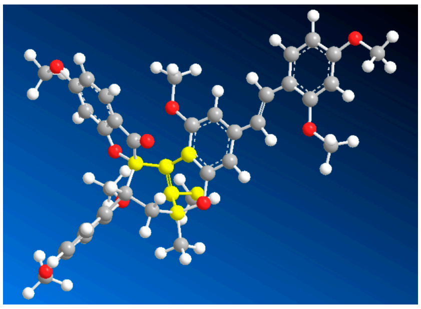

Figure SI-3h "wrong" structure and the twisted bond of **1b***

The 3D structure was optimized by MM2 field and the twisted double bond was shown in yellow color.

Table SI-1 HMBC correlationships of compound **1, 1a**, and **1b** (H→C)

No.	**1**	**1a**	**1b**
3	1, 2, 4, 5	1, 2, 4, 5	1, 2, 4
5	1, 3	1, 3	1, 2, 4
6	2, 4, α	2, 4, α	2, 4, α
α	2, 6, β, 1'	6, 1'	2, 6, 1'
β	1, α, 1', 2', 6'	1, 1', 2', 6'	1, 1', 2'
2'	β, 3', 4', 6'	β, 1', 3', 4'	β, 3', 4', 6'
6'	β, 2', 4', 5'	β, 2', 4', 5'	β, 2', 4', 5'
3"	3', 4', 5', 2",8"		
5"	1", 3", 4", 15", 16", 20"	1", 3", 8", 15"	1"
6"	*a*:4", 15"	*a*:5", 15"	*a*:5", 15"
	e: 1", 2", 4", 5", 7"	*e*:1", 2"	*e*:1", 2", 4"
7"	1", 2", 6"	1", 2", 6"	1", 2", 6"
11"	9", 10", 12"	9", 10", 12", 13"	9", 10", 12", 13"
13"	9", 11"	9", 11", 12"	9", 11"
14"	8", 9", 10", 12"	8", 10", 12"	8", 10", 12"
17"	16", 18"	15", 16", 18", 19"	15", 16", 18", 19"
19"	18"	15"	15", 17"
20"	5", 16", 18", 19"	5", 16", 18"	5", 16", 18"
OH/OMe-2	1, 2, 3	2	2
OH/OMe-4	3, 4, 5	4	4
OH/OMe-3'	2', 3', 4'	3'	3'
OH/OMe-5'		5'	
OH/OMe-2"	1", 2", 3"		2"
OH/OMe-10"		10"	
OH/OMe-12"		12"	12"
OH/OMe-16"			16"
OH/OMe-18"	17", 18", 19"	18"	18"
